# Current Knowledge and Future Perspectives on Mesenchymal Stem Cell-Derived Exosomes as a New Therapeutic Agent

**DOI:** 10.3390/ijms21030727

**Published:** 2020-01-22

**Authors:** Hyeon Su Joo, Ju Hun Suh, Hyeon Ji Lee, Eun Song Bang, Jung Min Lee

**Affiliations:** School of Life Science, Handong Global University, Pohang 37554, Korea; mecanum0342@gmail.com (H.S.J.); koreanjuu@naver.com (J.H.S.); peng9709@gmail.com (H.J.L.); 21500310@handong.edu (E.S.B.)

**Keywords:** mesenchymal stem cell, exosome, pre-conditioning, translational medicine

## Abstract

Mesenchymal stem cells (MSCs) are on the cusp of regenerative medicine due to their differentiation capacity, favorable culture conditions, ability to be manipulated in vitro, and strong immunomodulatory activity. Recent studies indicate that the pleiotropic effects of MSCs, especially their immunomodulatory potential, can be largely attributed to paracrine factors. Exosomes, vesicles that are 30-150 nanometers in diameter that function in cell-cell communication, are one of the key paracrine effectors. MSC-derived exosomes are enriched with therapeutic miRNAs, mRNAs, cytokines, lipids, and growth factors. Emerging evidences support the compelling possibility of using MSC-derived exosomes as a new form of therapy for treating several different kinds of disease such as heart, kidney, immune diseases, neural injuries, and neurodegenerative disease. This review provides a summary of current knowledge and discusses engineering of MSC-derived exosomes for their use in translational medicine.

## 1. Introduction

Mesenchymal stem cells (MSCs) have generated enormous interest in the field of regenerative medicine due to their ability to undergo multilineage differentiation, their favorable characteristics for culture and manipulation in vitro, and their strong immunomodulatory activity [1]. MSCs regulate T and B cells through the production of immunosuppressive molecules, including indoleamine 2,3-dioxygenase (IDO), nitric oxide (NO), prostaglandin E2 (PGE2), transforming growth factor (TGF)-β, haem oxygenase 1 (HO1), leukemia inhibitory factor, programmed death-ligand 1, hepatocyte growth factor (HGF), and galectins [2,3,4,5,6,7,8,9,10]. Recent studies suggest that the therapeutic effects of MSCs, especially those that are immunomodulatory, can be largely attributed to paracrine effectors [11]. These studies showed that MSCs do not engraft and replace damaged tissues directly. Instead, the paracrine effectors secreted by these cells exert therapeutic effects. Among such effectors, exosomes are considered key [11], having strong anti-inflammatory potential [12]. Here, we will summarize important points about the characteristics, isolation, and therapeutic applications of MSC-derived exosomes, as well as methods to increase their therapeutic potential through pre-conditioning of MSC cultures. Finally, we will provide a perspective on the future of MSC-derived exosomes in translational medicine.

## 2. Exosome Biology

### 2.1. Exosome Biogenesis

**Figure 1:**
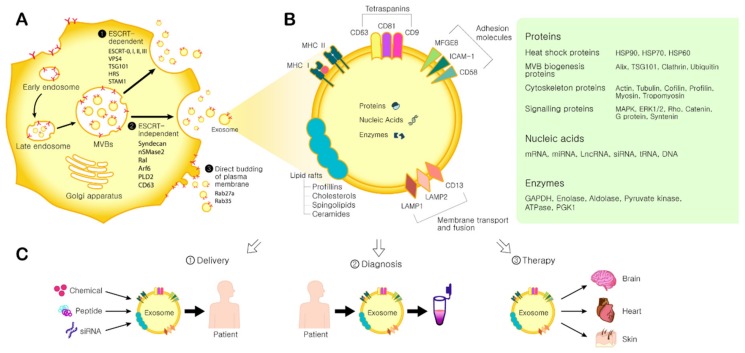
Exosome biogenesis and its application. (**A**) Exosome biogenesis. Three pathways for exosome biogenesis. (❶) ESCRT-dependent pathway and related proteins, (❷) ESCRT-independent pathway and related proteins, (❸) Direct budding of plasma membrane and related proteins. (**B**) Exosome components. MFGE8: milk fat globule-EGF factor 8 protein; ICAM-1: intercellular adhesion molecule 1; LAMP1,2: lysosomal-associated membrane protein 1,2; MHC I, II: major histocompatibility complex I, II. MAPK: mitogen-activated protein kinase; ERK: extracellular signal-regulated kinase; GAPDH: glyceraldehyde 3-phosphate dehydrogenase; PGK1: phosphoglycerate kinase 1. (**C**) Applications of exosomes. (①) Drug delivery: Therapeutic agents such as chemicals, peptides, and siRNAs can be delivered into patients. (②) Diagnosis: Exosomes derived from patients can be used for disease diagnosis. (③) Therapy: Exosomes derived from MSCs can be used to treat several diseases.

Cells secrete various types of extracellular vesicles (EVs). First discovered in 1967 by Peter Wolf, they were originally named “platelet dust” [13]. EVs are now categorized according to their diameter: apoptotic bodies, >1000 nm; microvesicles, 100–1000 nm; and exosomes, 30–150 nm [14]. The overlapping size of exosomes and microvesicles can be distinguished by their distinctive biogenesis pathways [15]. Exosomes are found in various physiological fluids, such as blood, saliva, urine, amniotic fluid, milk, and so on [16]. Exosome biogenesis occurs in early endosomes via the inward budding of the endosomal membrane to form intraluminal vesicles (ILVs), generating multivesicular bodies (MVBs) (Figure 1). Although endosome-dependent pathway is considered as a main biogenesis of exosomes, some studies suggest that direct budding of the plasma membrane also accounts for a significant portion of exosome biogenesis [17] (Figure 1). Upon fusion of MVBs with the plasma membrane, the ILVs are secreted extracellularly as exosomes. Several Rab GTPases (RAB11, RAB35, RAB27a, and RAB27b) mediate the fusion process [18]. The secreted exosomes are then taken up by recipient cells. Exosomes can be endocytosed or interact with recipient cells by ligand-receptor binding or direct binding [19].

The Endosomal Sorting Complexes Required for Transport (ESCRT) machinery functions to promote the budding and release of ILVs into the endosomal lumen, and thus exosome biogenesis (Figure 1). Each ESCRT complex consists of multiple proteins. ESCRT 0 recognizes and sequesters ubiquitinated proteins in the endosomal membrane, and recruits ESCRT I and II. Ubiquitin (Ub) is known as a signal molecule that sends membrane proteins or damaged cellular components to the lysosome for degradation [20]. It also acts as a signal for exosomal cargo sorting on the endosome membrane. MVB can either fuse with lysosomes for degradation or fuse with the plasma membrane to release exosomes [21]. Then, ESCRT I and II initiate intraluminal membrane budding by binding to the outer surface of the endosomal membrane near the ubiquitinated protein cargos, thereby selecting them to be in the newly-forming intraluminal buds in the MVB and serving an important role in cargo sorting. ESCRT III completes the process by sequestrating MVB proteins. After ILVs are generated, ESCRT III is separated from the MVB membrane by the sorting protein VPS4.

However, it has been reported that blocking the ESCRT pathway does not inhibit MVB formation, suggesting the possibility of an ESCRT-independent pathway (Figure 1) [22]. As one example of an alternative mechanism, the syndecans (SDC1-4), which are ubiquitous transmembrane proteins, directly regulate the ILVs during exosome formation by co-accumulating with syntenin and ALIX in exosomes. In this syndecan-syntenin-ALIX pathway, heparinase, ADP ribosylation factor 6, syndecan heparan sulfate proteoglycans, phospholipase D2, and syntenin mediate exosome budding [18]. There are other mechanisms in exosome biogenesis, indicated by the finding that sphingolipid ceramide is required for ILV formation. Neutral sphingomyelinase (nSMase) facilitates ILV formation by promoting MVB budding. In this pathway, exosomes are enriched with proteolipoprotein, CD63, CD81, and TSG101.

### 2.2. Isolation Methods for Exosomes

The conventional exosome isolation method is differential ultracentrifugation, which separates exosomes by density and size [23]. This method is easy, cost-effective, and advantageous when isolating exosomes from large volumes of biological fluids. However, this method lacks specificity, so the exosomes could be contaminated with other extracellular vesicles with similar diameters. To increase the purity of isolated exosomes, differential ultracentrifugation is used in conjunction with iodixanol or sucrose cushions [24,25]. Exosomes can also be isolated by filtration [26]. After cell debris and large molecules are removed, the filtrate can be ultrafiltered to eliminate small contaminants. Filtration methods can reduce the time and effort needed for isolation. However, because exosomes are isolated based on the pore size of the filter, it is hard to separate exosomes from contaminants like apoptotic bodies, or microbubbles that have similar diameters [27].

Immunoaffinity chromatography can be used to increase the purity of isolated exosomes. In this approach, antibodies in the column capture exosomes by binding to specific surface ligands of exosomes [28]. However, because only small sample volumes can be loaded at once, immunoaffinity chromatography is inappropriate for purifying exosomes from large sample volumes. Like the filtration method, size exclusion chromatography isolates exosomes by size [29], but uses beads that have pores within which exosomes can be captured. Large particles that cannot be captured by the beads pass through the column. Precipitation can be used to concentrate isolated exosomes. However, because chemical treatments can damage exosomes, or co-precipitate other molecules, precipitated exosomes cannot be used for further functional applications [30]. Although each isolation method has its own pros and cons, their limitations can be moderated in part by using the methods combinatorially, improving both yield and purity. Table 1 provides a summary and comparison of exosome isolation methods.

### 2.3. Exosome Components and Function

Because they form by budding from early endosomes, exosomes are encapsulated by a lipid bilayer membrane. Exosome membranes contain large amounts of cholesterol, sphingomyelin, and ceramide, which are present in lipid rafts [31]. Some studies suggest that certain lipid components, such as phosphatidylserine and prostaglandins, may play an important role in exosome function [32,33]. ExoCarta, an exosome database contains 41,860 protein, 1116 lipid molecule, 3408 mRNA, 2838 miRNA entries, derived from studies of exosomes in several species [34]. EVpedia, another data repository site, contains information about 92,897 proteins, 27,642 mRNAs, 4934 miRNAs, and 584 lipids, derived from 538 reports covering 33 species [35]. The most common proteins are members of the tetraspanin family, a group of scaffolding membrane proteins that include CD63, CD81, and CD9; they localize at the exosome surface and serve as markers [36]. Other common proteins found in these databases include membrane transporters and fusion proteins such as GTPases, annexins, and flotillin, heat shock proteins such as HSP70, MVB biogenesis proteins such as Alix and TSG101, lipid-related proteins, and phospholipases [37,38]. Exosomes also contain mRNAs and miRNAs. When exosomes are endocytosed by recipient cells, the genetic information carried by the RNAs can affect protein expression in those cells [39,40].

Depending on their specific characteristics, exosomes can be used in disease diagnosis, for drug delivery, and as therapeutic agents (Figure 1) [41]. Because exosomes contain unique bioactive molecules representing the components, physiological status, and character of the original cell [42], they have been referred to as the “fingerprint” of the parent cell [43]. For example, MHC II is found in EVs secreted by antigen presenting cells [44,45]. Also, several studies have confirmed that exosomes produced by cancer cells contain miRNAs that are identical to those in the original cancer cells. In addition, exosomes have a structure similar to that of liposomes and therefore their cargos are protected from the external environment, maintaining the integrity of cargo information. Moreover, because exosomes are found in body fluids, they can be isolated from patients in a non-invasive manner [46]. Altogether, these features support the idea that exosomes could be used as diagnostic biomarkers for cancer and neurodegenerative, metabolic, and infectious diseases, useful for defining the patient’s state of health [47] and helping clinicians make reliable diagnoses. It is of course important that the correlation between the disease and the biomarker be carefully confirmed. Other features of exosomes make them an attractive potential drug delivery tool [48]. Exosomes have the ability to mediate intercellular communication between widely separated locations in the body [49], moving throughout the body via the blood, and avoid inducing immune responses. Of great interest for drug delivery, exosomes can penetrate the blood-brain barrier [50,51]. Moreover, during migration, RNAs carried by exosomes are resistant to degradation by RNase activity. Finally, exosomes can be used as an alternative to cell therapy. Compared to therapies involving the injection of live cells that could cause uncontrolled cell growth and tumor formation, exosomes are safer and easier to control [52]. In contrast to cells, exosomes do not become mutated, do not duplicate, and cannot induce metastasis. In this review, we will focus on the therapeutic applications of MSC-derived exosomes and will describe it in detail below.

## 3. Therapeutic Applications of MSC-Derived Exosomes

Administration of MSC-derived exosomes has been shown to ameliorate disease phenotypes in a variety of disease models. For example, Teng et al. reported that in a rat myocardial infarction (MI) model, MSC-derived exosomes significantly improved tube formation by endothelial cells, impaired T cell functions, reduced infarct size, and preserved cardiac systolic and diastolic performance [53]. Furthermore, exosomes enriched with microRNA (miR)-11 by ischemic pre-conditioning were shown to significantly reduce infarct size and cardiac fibrosis by targeting methyl CpG binding protein 2 in a mouse post-myocardial infarction model [54]. Zhou et al. showed that exosomes from human umbilical cord-derived MSCs significantly reduced levels of blood urea nitrogen and creatinine, necrosis of proximal kidney tubules, and formation of abundant tubular protein casts by anti-oxidation and anti-apoptosis mechanisms in a cisplatin-induced acute kidney injury model [55]. In the lung, MSC-derived exosomes relieved hyperoxia-associated inflammation, bronchopulmonary dysplasia, pulmonary hypertension, fibrosis, and pulmonary vascular remodeling by modulating the lung macrophage phenotype in a preclinical model [56]. Therapeutic effects of exosomes in allergic conditions and wound healing processes have also been reported. Cho et al. showed that exosomes from human adipose tissue-derived MSCs reduced levels of lgE, eosinophils, infiltrated mast cells, and CD86^+^ and CD206^+^ cells in a mouse model of atopic dermatitis [57]. Umbilical cord MSC-derived exosomes prevented scar tissue formation and reduced myofibroblast accumulation by inhibiting the TGF-β/SMAD2 pathway in a skin-defect mouse model [58]. Umbilical cord MSC-derived exosomes also accelerated re-epithelialization with increased expression of CK19, PCNA, and collagen I in vivo [59]. In muscle tissue, MSC-derived exosomes enhanced regeneration by promoting myogenesis and angiogenesis; miR-494 is involved in this muscle regeneration process [60]. In work related to autoimmune conditions, Riazifar et al. demonstrated that MSC-derived exosomes reduced levels of pro-inflammatory cytokines and promoted the induction of regulatory T cells (Tregs) in an experimental autoimmune encephalomyelitis (EAE) model of multiple sclerosis [61].

Exosome treatment may also represent a new strategy for treating neural injuries and neurodegenerative diseases [62]. Exosomes enhanced functional recovery and increased neuroplasticity in young adult rats in which traumatic brain injury (TBI) had been induced [63]. In another TBI-related study, exosomes from bone marrow-derived MSCs diminished the lesion size, improved neurobehavioral performance, and exhibited neuroprotective effects by inhibiting the expression of pro-apoptosis protein Bcl-2-associated X protein, tumor necrosis factor (TNF)-α, and interleukin (IL)-1β, enhancing the expression of anti-apoptosis protein B-cell lymphoma 2, and regulating microglia/macrophage polarization [64]. In spinal cord injury-related studies, exosomes promoted angiogenesis, hindlimb locomotor activity, tissue sparing, reduced lesion size, endothelial cell proliferation, and attenuated inflammation and cellular apoptosis [65]. Exosomes regulated the actions of macrophages [66], and decreased neurotoxic A1 astrocytes activation via restraining nuclear translocation of NF_K_B p65 resulting in neuroprotection [67]. Ruppert et al. also reported similar results, which confirmed that MSC-derived EVs reduced inflammation with disorganized astrocytes and microglia in cord tissue resulting in improved locomotor activity [68]. Stroke is another complex disease that is a leading cause of death and disability. Emerging data show that exosomes exhibit neuroprotective effects after stroke, increasing nerve regeneration and neurological recovery and regulating peripheral immune responses; neurogenesis, angiogenesis, and axonal plasticity are improved [69]. In a study related to Alzheimer’s disease, the most prevalent neurodegenerative disease, MSC-derived exosomes stimulated neurogenesis and moderated beta amyloid (1–42)-induced cognitive impairment in mice [70]. Hypoxia-pre-conditioned MSC-derived exosomes ameliorated cognitive decline by rescuing synaptic dysfunction and regulating inflammatory responses in APP/PS1 mice, a model of Alzheimer’s disease [71]. These results suggest that treatment with MSC-derived exosomes could improve disease phenotypes in human patients with neurodegenerative conditions. Current clinical trials of exosomes are summarized in Table 2 (clinicaltrial.gov).

## 4. Pre-Conditioning Approaches to Enhance the Therapeutic Efficacy of Exosomes

As described above, MSC-derived exosomes exert therapeutic effects in various disease models. Because exosome characteristics vary depending on the status of the MSCs from which they are derived, and because the MSC status changes in response to external stimuli, several studies have investigated whether pre-conditioning MSCs can enhance the therapeutic activities of exosomes. Pre-conditioning of MSCs with cytokines, hypoxia, and chemicals has been shown to improve their immunosuppressive, immunomodulatory, and regenerative effects [73].

**Figure 2:**
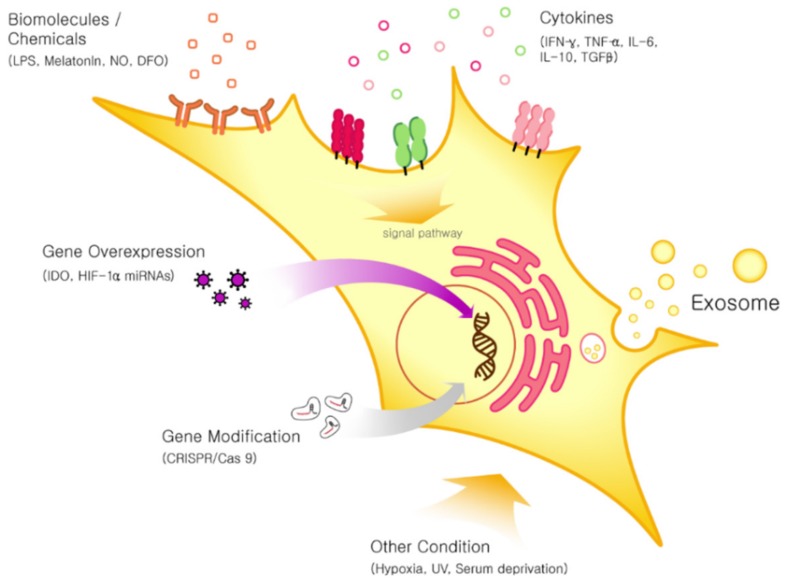
Pre-conditioning approaches for MSC to enhance the secretion and therapeutic efficacy of exosomes. LPS: lipopolysaccharides; NO: nitric oxide; DFO: deferoxamine; IFN: interferon; TNF: tumor necrosis factor; IL: interleukin; TGF: transforming growth factor; IDO: indoleamine-pyrrole 2,3-dioxygenase; HIF: hypoxia-inducible factors; miRNAs: micro RNAs; UV: ultraviolet.

Moreover, gene and cell surface modification of MSCs can enhance their therapeutic efficacy [74]. Pre-conditioning approaches that increase exosome activity directly, or indirectly by increasing MSC function, can both be utilized to maximize the therapeutic potential of MSC-derived exosomes (Figure 2).

### 4.1. Pre-Conditioning of MSCs: Increasing Exosome Production

Increasing the production of exosomes from MSCs is an urgent unmet medical need. Therefore, pre-conditioning approaches that increase exosome production are essential. One report showed that 3-D culture techniques had desirable effects. MSCs are usually cultured on 2-D surfaces in plastic plates, which lack conditions found in the MSC physiological niche. Use of a 3-D porous scaffold structure instead increased the production of exosomes from MSCs; furthermore, the exosomes had improved therapeutic effects in a rat brain disease model [75]. Large-scale MSC expansions by bioreactors and microcarriers can be utilized for increasing the production of exosomes [76]. Another approach indicated that vesiculation buffers containing chloride salts increased the production of EVs from Chinese hamster ovary (CHO) cells by osmotic stress [77]. Usage of 1- to 2-μm pore polymer filters improved the production of exosomes by extruding cells [78]. The production of EVs were also increased by treating cytochalasin B [79]. Interestingly, transplantation of exosomes from cytochalasin B-treated-MSCs showed angiogenic activities as in transplantation of MSCs [80]. Pre-conditioning using chemicals that stimulate the factors involved in exosome biogenesis may be an attractive approach. One study found that antifungal reagents increased exosome production in prostate cancer cells [81]. In this case, the modified imidazole, nitrefazole (20 μm), significantly increased exosome production from the C4-2B cell line. Nitrefazole increased the level of Rab27a, a protein that regulates MVB exocytosis. Treatment with nitrefazole and another azole, pentetrazole, significantly increased the levels of the exosome biogenesis-related molecules Alix and nSMase2. Such treatment also increased the p-ERK level. These data indicate that an azole might be a candidate for increasing exosome production from MSCs by modulating exosome biogenesis and release [81]. Gene editing might be another effective way to increase exosome production from MSCs. Various genes involved in exosome biogenesis and secretion could be modified to either overexpress the encoded proteins or knock out their functions [82]. For example, overexpression of phospholipase D2 led to a two-fold increase in the number of exosomes [83]. Table 3 and Table 4 summarize information about small molecules and gene modifications, respectively, that have been shown to increase exosome production. Although the targets have been tested in cancer cell lines, it would be worthwhile to test the effects of these modifications on exosome production in MSCs as well.

### 4.2. Pre-Conditioning of MSCs with Cytokines

Recent studies revealed that pro-inflammatory cytokines such as interferon (IFN)-γ, TNF-α, IL-1β, IL-6, and TGF-β effectively enhance the therapeutic functions of MSCs. Pre-conditioning of MSCs with IFN-γ stimulates the cells (referred to as IFN-γ-primed MSCs), which produce exosomes enriched in anti-inflammatory proteins, neuroprotective proteins, and anti-inflammatory RNAs [61,100,101]. Exosomes derived from IFN-γ-primed MSCs relieved several symptoms of multiple sclerosis in an EAE mouse model. When activated peripheral blood mononuclear cells (PBMCs) were cultured with exosomes from IFN-γ-primed MSCs, PBMC proliferation and the level of pro-inflammatory cytokines (IL-6, IL-22) decreased. In contrast, the level of immunosuppressive cytokines such as IDO increased [61]. Another study tested EVs from IFN-γ-primed MSCs. When cultured with EVs from IFN-γ-primed MSCs, the phagocytic and bacterial killing activity of a THP-1 cell line was enhanced. Furthermore, EVs from IFN-γ-primed MSCs attenuate *Escherichia coli*-induced lung injury in an *E. coli*-induced pneumonia mouse model [102]. A recent study demonstrated that pre-conditioning of MSCs with TNF-α (TNF-α-primed MSCs) produced exosomes containing miR-146a, which inhibit activation of fibroblast and inflammatory responses in an urethral fibrosis mouse model, demonstrating the anti-inflammatory effects of miR-146a [103]. Additionally, exosomes from TNF-α-primed MSCs were found to improve the proliferation and differentiation of primary human osteoblasts by increasing the expression of Wnt-3a in vitro [104]. The effects of pre-conditioning of MSCs with IL-1β (IL-1β-primed MSCs) in vitro and in vivo have also been reported. Song et al. investigated the therapeutic effects of IL-1β-primed MSCs and the exosomes produced by these cells in a mouse model of sepsis and found that the cells increased the survival rate of this model. Furthermore, IL-1β-primed MSCs showed upregulated expression of anti-inflammatory miR-146a; exosomes from these cells also contained miR-146a, which was transferred to macrophages and induced M2 polarization [105]. Wang and coworkers demonstrated that pre-conditioning of MSCs with TGF-β (TGF-β-primed MSCs) increased the expression of miR-135b in exosomes, which promoted chondrocyte proliferation in vitro through Sp1 regulation and cartilage tissue repair in an osteoarthritis rat model [106].

Several studies have investigated the use of a combination of pro-inflammatory cytokines for MSC pre-conditioning. EVs from IFN-γ- and TNF-α-primed MSCs showed different protein, miRNA, and cytokine profiles compared to EVs from naïve MSCs. Culturing lipopolysaccharide (LPS)-induced rat splenocytes with EVs from IFN-γ- and TNF-α-primed MSCs markedly reduces the levels of pro-inflammatory cytokines (IFN-γ, TNF-α) in the splenocytes in vitro, an effect that is due to increased levels of prostaglandin E2 (PGE2) and cyclooxygenase 2 [101]. Another study showed that expression of several immunosuppressive factors (IDO, PGE2, IL-8, IL-6, CCL-2) was increased in IFN-γ- and TNF-α-primed MSCs. Moreover, exosomes from the primed MSCs showed increased levels of miR-34a and miR-146a, which are potential anti-inflammatory factors, skewing macrophage differentiation toward the M2 phenotype [100]. Another study showed that exosomes from IFN-γ- and TGF-β-primed MSCs promote mononuclear cell transformation into Tregs in vitro [106].

### 4.3. Pre-Conditioning of MSCs with Hypoxia

Hypoxia conditioning is a widely used approach for priming MSCs and MSC-derived exosomes. Several studies have demonstrated that exosomes from MSCs pre-conditioned with hypoxia (hypoxia-primed MSCs) promote angiogenesis relative to exosomes from MSCs cultured under normoxic conditions. In 2012, Zhang et al. reported that microvesicles from hypoxia-primed human umbilical cord (UC) MSCs promoted UC-endothelial cell (UC-EC) proliferation in vitro and also improved blood flow recovery in a hindlimb ischemia rat model [107]. Salomon et al. investigated the effects of exosomes from hypoxia-primed placental MSCs. These exosomes increased migration and promoted tube formation by placental microvascular endothelial cells [108]. Exosomes from hypoxia-primed MSCs increased migration and capillary network formation by human umbilical vein ECs (HUVECs) in vitro. In addition, exosomes from hypoxia-primed MSCs attenuated inflammation after fat grafting, increased fat survival [109]. Co-transplantation of exosomes with a fat graft resulted in increased expression of EGF, fibroblast growth factor, angiopoietin-1, and angiopoietin receptor (Tie-1) proteins [110]. It was observed that exosomes from hypoxia-primed MSCs versus MSCs cultured under normoxic conditions were more easily taken up by HUVECs; the uptaken exosomes promoted vascular endothelial growth factor (VEGF) expression and protein kinase A signaling pathway activation, which resulted in improved angiogenesis by HUVECs [111,112]. Exosomes from hypoxia-primed MSCs have also been shown to promote myocardial repair. One study found that exosomes transferred miR-210 to HUVECs in an nSMase2-dependent manner, resulting in improved angiogenic and anti-apoptotic functions of HUVECs in vitro and cardioprotection (improved vascularization and survival rate) in an MI mouse model [113]. Exosomes from hypoxia-primed MSCs also contain miR-125b, which impedes cell death in the MI mouse model [113]; another study suggested that the exosomes directly suppress GSK3β expression through miRNA-26a, resulting in cardioprotective effects in an MI rat model [114].

Exosomes from hypoxia-primed MSCs can also promote regeneration, alter immune responses, and exert neuroprotective effects. For example, medium from adipose-derived, hypoxia-primed MSCs contains more cytokines (IL-6, TNF-α) and growth factors (HGF, VEGF) and promotes AML12 cell proliferation compared to normoxic condition through JAK/STAT3 signaling in vitro [115]. Furthermore, microvesicles derived from hypoxia-primed MSCs were found to promote cell proliferation and migration of U2OS cells in vitro, partially through the PI3K/AKT and HIF-1α pathway [116]. Exosomes from hypoxia-primed MSCs also have immunomodulatory functions [117]. In an endotoxin-induced acute lung injury (ALI) mouse model, exosomes from hypoxia-primed MSCs reduced the level of white blood cells and neutrophils in the bronchoalveolar lavage (BAL) fluid. Levels of MIP-2 (also known as CXCL2) and osmotic protein in BAL also deceased [96]. It has been reported that hypoxia-primed MSCs go through glycolytic reprogramming, which results in the production of anti-inflammatory exosomes and is related to M2 macrophage polarization and Treg induction [118]. Another study suggested that M2 polarization is partially mediated by miR-21-5p contained in exosomes [119]. Finally, in an APP/PS1 mouse model of Alzheimer’s disease, exosomes from hypoxia-primed MSCs decreased amyloid plaque deposition and the beta amyloid level, and upregulated the expression of synaptic proteins such as growth-associated protein 43 and synapsin 1 [71]. Exosomes from hypoxia-primed MSCs inhibited astrocyte and microglia activation and promoted the transformation of microglia to dendritic cells, resulting in a neuroprotective effect in vivo. Moreover, the exosomes decreased the expression of pro-inflammatory cytokines (TNF-α, IL-1β) and increased that of anti-inflammatory cytokines (IL-4, IL-10).

### 4.4. Pre-Conditioning of MSCs with Biomolecules or Chemicals

Some studies have compared the effects of exosomes from MSCs pre-conditioned with LPS (LPS-primed MSCs) with exosomes from unconditioned MSCs. The secretome of such MSCs has been shown to improve liver regeneration by decreasing serum IL-6 and TNF-α levels in vivo [120]. Other studies revealed that exosomes from LPS-primed MSCs induced THP-1 to increase production of anti-inflammatory cytokines and promote M2 polarization [121]. The miRNA let-7b, which is involved in monocyte activation and differentiation, is present at especially high levels in these exosomes. A recent study has also shown that culturing macrophages with exosomes from LPS-primed MSCs increases *STAT3* gene expression, secretion of cytokines (IL-10 and IL-15), and expression of growth factors (FLT-3L); such macrophages also extend survival in a mouse model of acute radiation syndrome [122]. A different study showed that exosomes from LPS-primed MSCs increased M2 macrophage polarization, resulting in the attenuation of post-infarction inflammation and cardiomyocyte apoptosis in a mouse model of MI [123].

A variety of other molecules have been tested as pre-conditioning agents. Recent studies have examined the effects of thrombin. Exosomes from MSCs pre-conditioned with thrombin (thrombin-primed MSCs) improved proliferation, migration, and tube formation by HUVECs in vitro and cutaneous wound healing in vivo [124]. In another study, exosomes from thrombin-primed MSCs from Wharton’s Jelly showed enhanced anti-inflammatory and anti-apoptotic effects, resulting in attenuation of brain infarction in a rat model of hypoxic ischemic encephalopathy [124]. Exosomes from MSCs pre-conditioned with melatonin decreased the expression of *HIF1α*, *ICAM1*, *IL1B* and *NFkB*, and increased the expression of *BCL2*, *HO1*, *IL10* and *VEGF* in a rat model of renal ischemia-reperfusion injury [125], whereas exosomes from MSCs pre-conditioned with advanced glycation end products contained a high level of miR-146a and inhibited calcification of vascular smooth muscle cells in vitro [126]. One study reported that pre-conditioning MSCs with deferoxamine increased the expression of miR-126a; exosomes derived from these cells promoted angiogenesis by HUVECs and promoted cutaneous wound healing in a diabetic rat model [127]. Interestingly, treating MSCs with Suxiao Jiuxin pill (a traditional Chinese medicine used in acute coronary syndrome) enhanced the function of derived exosomes, which increased the proliferation of cardiomyocytes by decreasing the expression of the H3K27 demethylase UTX [128]. Exosomes from MSCs pre-conditioned with NO contained an increased level of VEGF and miR-126, and promoted angiogenesis by HUVECs [129]. In another study using sodium nitroprusside (SNP) as an NO donor, SNP-primed MSC microvesicles, which were enriched with Jagged-1 and *Vegf-A* mRNAs, were found to improve the transplantation efficacy of hematopoietic stem cells by enhancing Jagged-1 and Vegf-A expression [130]. In addition, exosomes from MSCs pre-conditioned with H_2_O_2_ increased the proliferation and migration of HUVECs and improved skin flap recovery in a rat model of ischemia-reperfusion injury [131].

### 4.5. Gene Overexpression to Improve the Function of MSC-Derived Exosomes

Several studies have investigated the effects of gene overexpression on MSC-derived exosomes. In 2010, one study reported that paracrine factors from GATA-4-overexpressing MSCs increased the angiogenic function and survival of HUVECs [132]. It was then demonstrated that exosomes from such MSCs improved cardiac function (increasing the number of heart vessels and decreasing the number of apoptotic cardiac cells) in an MI mouse model [133]. GATA-4-overexpressing MSCs were shown to contain cardioprotective, anti-apoptotic miR-19a, which activates the Akt and ERK signaling pathways [134]. Likewise, exosomes secreted by CXCR4-overexpressing MSCs promoted tube formation by HUVECs and exerted a cardioprotective effect through the Akt signaling pathway in an MI rat model [135]. In another study, exosomes derived from MSCs overexpressing Akt were found to promote angiogenic functions in vitro and improve cardiac regeneration by activating platelet-derived growth factor D in an acute MI rat model [136]. It is known that IDO mediates immunomodulatory effects of MSCs [137], and exosomes derived from IDO-overexpressing rat MSCs were found to increase the number of Tregs, decrease the number of CD8^+^ T-cells and levels of pro-inflammatory cytokines, and promote immune tolerance in cardiac allografts [138]. Exosomes from MSCs overexpressing stromal-derived factor 1a, which is involved in cardiac repair, were shown to inhibit myocardial cell apoptosis and promote regeneration of the cardiac endothelial microvascular in vivo [139].

Overexpression of miRNAs in MSCs can also enhance the cardioprotective function of exosomes. For example, exosomes derived from miR-214-overexpressing MSCs protected cardiac stem cells from oxidative damage by silencing CaMKII [140]. MSCs overexpressing miR-133 produced exosomes that decreased inflammation and myocardial fibrosis in an acute MI rat model [141]. Furthermore, MSCs that overexpressed miR-93-5p and miR-146 produced exosomes that attenuated MI-induced myocardial damage by suppressing expression of inflammatory cytokines and hypoxia-induced autophagy in vitro [140], and by decreasing expression of early growth response factor 1 in vivo [142]. Exosomes derived from miR-126-overexpressing MSCs also reduced hypoxia-induced expression of inflammation factors and promoted microvascular generation and myocardial cell migration in vitro; these exosomes also decreased cardiac fibrosis and the level of inflammatory cytokines in vivo [143].

Overexpression of certain genes in MSCs has been observed to increase the neuro-protective and -regenerative activity of the exosomes produced by these cells. In 2013, Xin et al. suggested that exosomes derived from miR-133b-overexpressing MSCs increased axon plasticity and neurite remodeling in the ischemic boundary zone in a rat model of middle cerebral artery occlusion (MCAO) [144]. These authors also demonstrated that astrocytes treated with exosomes derived from miR-133b-overexpressing MSCs increased neurite branching, and elongation of cortical embryonic rat neurons in vivo [145]. Moreover, exosomes from miR-133b-overexpressing MSCs also suppressed *RhoA* expression and activated ERK1, ERK2, and CREB in brain tissues in an intracerebral hemorrhage rat model, which resulted in attenuation of apoptosis and neurodegeneration [146]. In other experiments, exosomes from miR-30d-5p-overexpressing MSCs suppressed autophagy by promoting M2 polarization of microglia and macrophages, which reversed brain injury induced by acute ischemic stroke and autophagy in an MCAO mouse model [140]. Finally, exosomes from miR-25-overexpressing MSCs decreased levels of IL-1β and TNF-α and increased the number of motor neurons in a rat model of transient ischemia, resulting in spinal cord protection [147].

The consequences of gene overexpression on the anti-tumor effects of MSCs have also been investigated. Exosomes from MSCs expressing TRAIL induced apoptosis in cancer cells in vitro [148]. A different study showed that exosomes derived from miR-122-overexpressing MSCs sensitize hepatocellular carcinoma (HCC) to chemotherapeutic agents (sorafenib), enhancing the antitumor activity of sorafenib in vitro and in vivo [149]. Another study reported that exosomes from MSCs that overexpress a small interfering RNA against GRP78, which is overexpressed in sorafenib-resistant cancer cells, re-sensitize HCC to sorafenib in vitro and in vivo [150]. Exosomes from miR-119a-overexpressing MSCs suppressed the proliferative, invasive, and migrative activities of glioma cells, resulted in suppression of glioma progression by downregulating ankyrin repeat and PH domain 2 in vitro [151]. Exosomes from miR-16-5p-overexpressing MSCs inhibit the proliferation, migration, and invasion of colorectal cancer cells (CRCs) and promote the apoptosis of such cells by decreasing the expression of integrin α2 in vitro [28]. Proliferation of glioma stem cell line is reduced by treatment with exosomes from miR-124a-overexpressing MSCs in vitro [152]. Furthermore, treatment of mice bearing implanted GSC267 glioblastoma cells with exosomes from miR-124a-overexpressing MSCs improves the survival rate of the mice [152]. In other work, the migration and invasion of prostate cancer cells was shown to be inhibited in vitro by exosomes from miR-143-overexpressing MSCs via downregulation of trefoil factor 3 [153]. In another study, the proliferative, migrative, and invasive activities of oral cancer cells were suppressed by exosomes from miR-101-3p-overexpressing MSCs via targeting of collagen type X alpha 1 chain [154].

Gonzalez-King et al. demonstrated that exosomes from HIF-1α-overexpressing MSCs improved tube formation by HUVECs and induced new vessel formation in vivo through a Jagged 1-dependent mechanism [155]. Some studies have reported that exosomes derived from genetically modified MSCs have a bone regenerative function. Exosomes from miR-140-overexpressing MSCs increased the proliferative and migrative activities of articular chondrocytes in vitro and attenuated symptoms of osteoarthritis (OA) in vivo [156]. Other research showed that exosomes from miR-92a-3p-overexpressing MSCs promoted chondrogenesis in vitro and suppressed cartilage degradation in an OA mouse model by repressing *WNT5A* expression [157]. Recent studies showed that exosomes from miR-375-overexpressing MSCs promoted osteogenic differentiation in vitro by inhibiting the expression of insulin-like growth factor binding protein 3 and improved bone regeneration in a calvarial defect rat model [158].

Gene overexpression has also been shown to enhance the therapeutic effects of MSC-derived exosomes in models of liver and lung diseases. Exosomes from miR-181-5p-overexpressing MSCs downregulated collagen I, vimentin, and fibronectin, and showed an anti-fibrotic function in a liver fibrosis mouse model [159]. Exosomes from miR-223-overexpressing MSCs downregulated NLRP3 and caspase-1, and exhibited protective effects in injured hepatocytes and an experimental autoimmune hepatitis model [160]. Another study showed that exosomes from miR-20a-overexpressing MSCs downregulated beclin-I and FAS, and alleviated apoptosis and autophagy in a liver ischemia-reperfusion rat model [161]. In LPS-induced alveolar epithelial cells, exosomes from miR-30b-3p-overexpressing MSCs promoted cell proliferation and diminished cell apoptosis by downregulating SAA3. Such exosomes also showed protective effects in an ALI mouse model [162]. Tal et al. reported that exosomes from miR-126-3p-overexpressing MSCs stimulated angiogenesis and collagen maturity in a diabetic rat model [156]. Exosomes from miR-125b-overexpressing MSCs were reported to repress *Myo1e* expression and suppress the proliferation and migration of vascular smooth muscle cells in vitro and in vivo, suppressing neointimal hyperplasia [163].

## 5. Conclusions

The number of exosome-related manuscripts, and the funding to support this work, are exponentially growing, representing enthusiasm about this area of research [164]. In particular, MSC-derived exosomes are being extensively investigated as potential treatments for various intractable diseases (Table 2). MSC-derived exosomes have captured attention as possible therapeutic agents because they carry most of the therapeutic effect of the MSCs themselves. Moreover, exosomes are a cell-free therapy, which would minimize safety concerns about injecting live cells. The therapeutic efficacy of MSC-derived exosomes has been shown in heart, kidney, lung, skin, muscle, and brain diseases. In many cases, the mode of action of such exosomes in treating diseases is derived from their anti-inflammatory activity. MSC-derived exosomes contain anti-inflammatory components, which are delivered to the recipient cells, reducing inflammation. Taking advantage of these characteristics, MSC-derived exosomes can be applied to various inflammatory diseases and autoimmune diseases. Interestingly, exosome activity can be easily manipulated by pre-conditioning of MSCs, simply by adding cytokines or chemicals into the culture medium, introducing gene modifications, or using hypoxic culture conditions. CRISPR/Cas9, a recent genome editing technology also can be applied to improving the therapeutic efficacy of MSC-derived exosomes (Figure 2) [165].

Many factors and concerns should be resolved before MSC-derived exosomes are applied clinically. First, standards for exosome purity should be established. To date, vesicle size and expression of markers (CD63, CD9, CD81) have been used to identify isolated exosomes. However, in some cases, these methods can result in contaminated mixtures. Therefore, guidelines for purity and levels of acceptable contamination of isolated exosomes are necessary. Second, standards for quality control (QC) of isolated MSC-derived exosomes should be established. MSCs have different physiological states, which could affect the therapeutic efficacy of derived exosomes [166]. The issue could be addressed in part by using pre-conditioning or MSCs derived from induced pluripotent stem cells or embryonic stem cells [167], because those approaches could minimize the lot-to-lot variation found for primary naive MSCs. Defining markers for QC will also be helpful. If specific miRNAs, peptides, or cytokines could be used as reliable indicators, then QC for isolated exosomes would be robust. Taken together, results from a large body of work indicate that MSC-derived exosomes have tremendous potential as therapeutic agents for treating several intractable diseases, especially inflammatory conditions. Efforts to establish guidelines and standards for efficacy and safety issues in conjunction with pre-conditioning approaches for the therapeutic effects of exosomes will accelerate clinical applications of MSC-derived exosomes.

## Figures and Tables

**Table 1:** Summary of exosome isolation methods.

Methods	Principles & Materials	Advantages	Disadvantages	Downstream Applications
Ultracentrifugation	Physical method	-Low cost -Allows purification from large volumes -Keeps exosomes intact	-Low yield -Low purity	-Analysis of nucleic acids, biomarkers -Encapsulation of cargos
Ultrafiltration	Physical method using filters	-Reduced time/effort -High yield -Keeps exosomes intact	-Low purity -Deformation of exosomes	-Analysis of nucleic acids, biomarkers
Immunoaffinity chromatography	Chemical method using antibodies	-High yield -High purity	-Not compatible with large volumes -Time-consuming -High cost	-Analysis of nucleic acids, proteins
Size exclusion chromatography	Physical/chemical method using columns packed with pore beads	-High yield -Reduces exosome aggregation -Keeps exosomes intact	-Samples can be diluted -Time-consuming	-Analysis of nucleic acids, biomarkers, proteins -Encapsulation of cargos
Precipitation	Physical/chemical method	-High yield -Easy -Concentrates diluted samples	-Post-clean up is needed for downstream applications	-Analysis of nucleic acids, biomarkers

**Table 2:** Current clinical trials of exosomes. cGVHD: choronic Graft versus Host Disease.

NCT Numbers	Years	Conditions	Sources	Country	Group	Features
NCT01159288	2010	Non-Small Cell Lung Cancer	Dendritic Cell	France	Institut Gustave Roussy	Exosome as adjuvant [72]
NCT01294072	2011	Colon Cancer	Plant	USA	James Graham Brown Cancer Center	
NCT01668849	2012	Head and Neck Cancer, Oral Mucositis	Plant	USA	James Graham Brown Cancer Center	
NCT02138331	2014	Type 1 Diabetes	MSC	Egypt	Sahel Teaching Hospital	
NCT02565264	2015	Cutaneous Ulcer	Plasma	Japan	Kumamoto University	
NCT03608631	2018	Metastatic Pancreatic Adenocarcinoma	MSC	USA	M D Anderson Cancer Center	Exosomes with KRAS G12D siRNA
NCT03437759	2018	Macular Holes	MSC	China	Tianjin Medical University Hospital	
NCT03384433	2019	Cerebrovascular Disorders	MSC	Iran	Shahid Beheshti University of Medical Sciences	Exosome enriched by miR-124
NCT04202783	2019	Craniofacial Neuralgia	-	USA	Neurological Associates of West LA	Exosome as delivery vehicle
NCT04202770	2019	Depression, Anxiety, Dementias	-	USA	Neurological Associates of West LA	Exosome as delivery vehicle
NCT03493984	2019	Polycystic Ovary Syndrome	Plant	USA	University of Louisville	
NCT04134676	2019	Chronic Ulcer	MSC	Indonesia	Mayapada Hospital, Indra Clinic, Sukma Cliniq	MSC-derived EV
NCT04213248	2020	Dry Eye in Patients with cGVHD	Umbilical MSC	China	Zhongshan Ophthalmic Center	

**Table 3:** List of small molecules and treatments that affect exosome release.

Small Molecule or Treatment	Cancer Cell Type
C6 ceramide	Multiple myeloma cells [84]
Hypoxia	Breast cancer cells [85]
Acidic pH/Protein pump inhibitors	Melanoma cells [86]
Tunicamycin	Cervical cancer cells [87]
Monensin	Leukemia cells [88]
Irradiation	Prostate cancer cells [89]
UV radiation	Colon cancer cells [90]
Doxorubicin	Prostate cancer cells [91]
Photodynamic treatment	Prostate cancer cells [91]
Melphalan	Multiple myeloma cells [92]
CI-1033/PF-00299804	Glioma cells [93]

**Table 4:** List of genes that are involved in exosome release.

Gene/Modification Method	Cancer Cell Type
Plkfyve/KD	Prostate cancer cells [94]
Cortactin/KD	HNSCC cells [95]
EGFR/oncogenic EGFRvIII expression	Glioma cells [93]
Ras/oncogenic HRas expression	Intestinal epithelial cells [96,97]
Liver Kinase B1/expression	Lung cancer cells [98]
ElF3C/overexpression	Liver cancer cells [99]

KD: knockdown; HNSCC: head and neck squamous cell carcinoma; EGFR: epidermal growth factor receptor; EIF3C: eukaryotic translation initiation factor 3 subunit C.
